# The human urinary proteome contains more than 1500 proteins, including a large proportion of membrane proteins

**DOI:** 10.1186/gb-2006-7-9-r80

**Published:** 2006-09-01

**Authors:** Jun Adachi, Chanchal Kumar, Yanling Zhang, Jesper V Olsen, Matthias Mann

**Affiliations:** 1 Department of Proteomics and Signal Transduction, Max-Planck Institute for Biochemistry, Am Klopferspitz, D-82152 Martinsried, Germany; 2 Center for Experimental Bioinformatics, University of Southern Denmark, Campusvej, DK-5230 Odense M, Denmark; 3 Current address: Graduate School of Global Environmental Studies, Kyoto University, Yoshida-Honmachi Sakyo-Ku, Kyoto, Japan; 4 Beijing Institute of Genomics, Chinese Academy of Sciences, Beijing 101300, China

## Abstract

**Background:**

Urine is a desirable material for the diagnosis and classification of diseases because of the convenience of its collection in large amounts; however, all of the urinary proteome catalogs currently being generated have limitations in their depth and confidence of identification. Our laboratory has developed methods for the in-depth characterization of body fluids; these involve a linear ion trap-Fourier transform (LTQ-FT) and a linear ion trap-orbitrap (LTQ-Orbitrap) mass spectrometer. Here we applied these methods to the analysis of the human urinary proteome.

**Results:**

We employed one-dimensional sodium dodecyl sulfate polyacrylamide gel electrophoresis and reverse phase high-performance liquid chromatography for protein separation and fractionation. Fractionated proteins were digested in-gel or in-solution, and digests were analyzed with the LTQ-FT and LTQ-Orbitrap at parts per million accuracy and with two consecutive stages of mass spectrometric fragmentation. We identified 1543 proteins in urine obtained from ten healthy donors, while essentially eliminating false-positive identifications. Surprisingly, nearly half of the annotated proteins were membrane proteins according to Gene Ontology (GO) analysis. Furthermore, extracellular, lysosomal, and plasma membrane proteins were enriched in the urine compared with all GO entries. Plasma membrane proteins are probably present in urine by secretion in exosomes.

**Conclusion:**

Our analysis provides a high-confidence set of proteins present in human urinary proteome and provides a useful reference for comparing datasets obtained using different methodologies. The urinary proteome is unexpectedly complex and may prove useful in biomarker discovery in the future.

## Abstract 2

A high confidence set of proteins in urine from healthy donors is described as a reference urinary proteome.

## Background

Urine is formed in the kidney by ultrafiltration from the plasma to eliminate waste products, for instance urea and metabolites. Although the kidney accounts for only 0.5% of total body mass, a large volume of plasma (350-400 ml/100 g tissue/min) flows into the kidney, generating a large amount of ultrafiltrate (150-180 l/day) under normal physiologic conditions [1,2]. Components in the ultrafiltrate such as water, glucose, amino acids, and inorganic salts are selectively reabsorbed, and less than 1% of ultrafiltrate is excreted as urine. Serum proteins are filtered based on their sizes and charges at the glomeruli [3]. After passing through glomeruli, abundant serum proteins such as albumin, immunoglobulin light chain, transferrin, vitamin D binding protein, myoglobin, and receptor-associated protein are reabsorbed, mainly by endocytic receptors, megalin, and cubilin in proximal renal tubules [4-8]. Thus, protein concentration in normal donor urine is very low (less than 100 mg/l when urine output is 1.5 l/day), and normal protein excretion is less than 150 mg/day. This is about a factor 1000 less compared with other body fluids such as plasma. Excretion of more than 150 mg/day protein is defined as proteinuria and is indicative of glomerular or reabsorption dysfunction.

Urine can be collected in large amounts fully noninvasively. Therefore, despite the low protein concentration, more than adequate amounts of material (at least 0.5 mg) can be collected from a single sample, although protein in urine must be concentrated. This advantage of urine as a body fluid for diagnosis also allows collection of samples repeatedly over lengthy time periods. Furthermore, normal urinary proteins generally reflect normal kidney tubular physiology because the urinary proteome contains not only plasma proteins but also kidney proteins [7,9-13]. Thus, urine is good material for the analysis of disease processes that affect proximal organs, such as kidney failure resulting from high blood pressure and diabetic nephropathy, which is the most frequent cause of renal failure in the Western world [14].

Urinary proteomics has been conducted by combining various protein concentration and protein separation methods as well as mass spectrometry (MS) technology. In many studies, two-dimensional gel electrophoresis was employed for protein separation. One of these studies, that conducted by Pieper and coworkers [11], identified 150 unique proteins using two-dimensional gel electrophoresis and both matrix-assisted laser desorption ionization time-of-flight MS and liquid chromatography (LC)-tandem mass spectrometry (MS/MS or MS^2^). However, one-dimensional and two-dimensional chromatographic approaches have been used in several recent studies, resulting in further protein identifications. Pisitkun and coworkers [9] reported identification of 295 unique proteins from the exosome fraction using one-dimensional gel electrophoresis and LC-MS/MS. Sun and colleagues [12] identified 226 unique proteins using one-dimensional gel electrophoresis plus LC-MS/MS and multidimensional liquid chromatography (LC/LC)-MS/MS. Wang and coworkers [13] applied concanavalin A affinity purification for the enrichment of N-glycoprotein in urine and identified 225 proteins using one-dimensional gel electrophoresis plus LC-MS/MS and LC/LC-MS/MS. Recently, Castagna and colleagues [10] exploited beads coated with a hexametric peptide ligand library for urinary protein concentration and equalization, and identified 383 unique gene products by LC-MS/MS using a linear ion trap-Fourier transform (LTQ-FT) instrument. These researchers combined their set of urinary proteins with others derived from the literature to yield a total of about 800 proteins.

Some of these five largest urinary proteome catalogues contain proteins with single peptide identification (>30% of total identified proteins reported by Pisitkun and coworkers [9]) and lack an assessment of false-positive ratios. Moreover, proteins identified in these studies seem to be the tip of the iceberg of the urinary proteome, because nearly 1000 protein spots separated by two-dimensional gel remain unidentified [11]. These studies suggest that three steps are especially important for deep analysis: protein concentration from urine with minimal loss; protein separation to reduce the complexity of the protein mixture and remove abundant proteins; and peptide sequencing with high mass accuracy and rapid scanning.

In the present study, we employed a simple and straightforward method, namely ultrafiltration, for protein concentration. For protein separation, one-dimensional gel electrophoresis or reverse phase column chromatography was used. For peptide sequencing, we employed methods recently developed in our laboratory involving the LTQ-FT and linear ion trap-orbitrap (LTQ-Orbitrap), which have extremely high mass accuracy [15,16]. The LTQ facilitates accumulation of a greater number of charges than is possible with traditional three-dimensional ion traps, and it is sufficiently fast to enable two consecutive stages of mass spectrometric fragmentation (MS/MS/MS or MS^3^) on a chromatographic time scale. The Fourier transform-ion cyclotron resonance (FTICR) part of the instrument provides a very high resolution of 100,000 and mass accuracies in the sub-ppm (parts per million) range using selected ion monitoring (SIM) scans. For complex protein samples, the LTQ-FT was shown to increase the number of high-confidence identifications compared with an LCQ instrument [17]. Together, high mass accuracy and MS^3 ^result in dramatically increased confidence for peptide identification [15] and allow 'rescue' of protein identifications by single peptides. A novel hybrid mass spectrometer, the LTQ-Orbitrap [18] also provides a high mass resolving power of 60,000 and high-accuracy mass measurements (sub-ppm on average) using a lock mass strategy, even without SIM scans [15].

These techniques enabled us to identify 1543 proteins in urine from an in-depth study from a single individual and pooled urine obtained from nine individuals, while virtually eliminating false-positive identifications. In the LTQ-FTICR dataset 337 proteins (26.3% of the total identified proteins) were identified with single unique peptide using MS^2 ^and MS^3^. Around a third of all characterized proteins are annotated as extracellular proteins. In the total data set we found 488 proteins to be annotated as membrane proteins (47% of all proteins with localization information). Of these proteins, 225 proteins were annotated as plasma membrane proteins (21.6%). These proteins include water, drug, sodium, potassium, and chloride transporters that are localized in the kidney and regulate homeostasis of body fluids. This high-confidence collection of proteins present in human urine can serve as a reference for future biomarker discovery.

## Results

### Identification of urinary proteins

**Figure 1:**
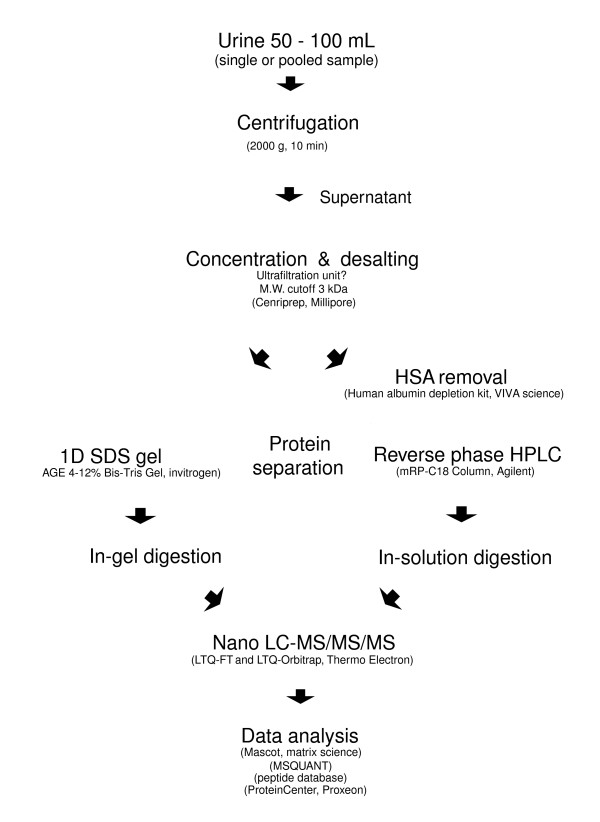
An overview of the procedure used for analysis of the urinary proteome. 1D, one-dimensional; HPLC, high-performance liquid chromatography; HSA, human serum albumin; MW, molecular weight; LC, liquid chromatography; MS, mass spectrometry; SDS, sodium dodecyl sulfate.

**Figure 2:**
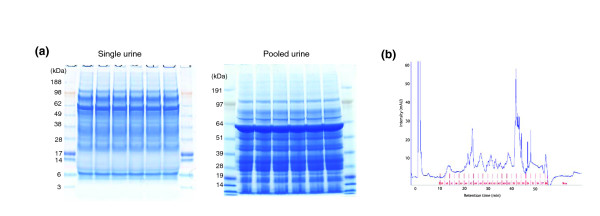
Urinary protein separation by one-dimensional SDS gel and reverse-phase HPLC. **(a) **150 μg urinary protein (25 μg/lane) from single sample and pooled sample were applied on a 4-12% Bis-Tris gel. Gel was stained by colloidal Coomassie and cut into 14 pieces (in-gel 1 set) or 10 pieces (in-gel 2 set) for single urine sample, and cut into 10 pieces for pooled urine sample. **(b) **250 μg of urinary protein was applied to Vivapure Anti-HSA Kit to deplete serum albumin. The albumin-depleted protein mixture was dissolved in 6 mol/l urea and 1.0% acetic acid solution, and separated on mRP-C18 High-Recovery protein column at 80°C using linear multi-segment gradient, as described in the Materials and Methods section. HPLC, high-performance liquid chromatography; SDS, sodium dodecyl sulfate.

Normal total protein concentration in urine is very low and usually does not exceed 10 mg/100 ml in any single specimen (normal protein excretion is less than 150 mg/day). To concentrate and de-salt urinary proteins, various sample preparation procedures such as ultrafiltration, centrifugation, reverse-phase separation, dialysis, lyophilization, enrichment of proteins by affinity column or beads, and precipitation using organic solvents have been used [9-13,19-21]. As shown in Figure 1, we used an ultrafiltration unit, because it allows us to concentrate and desalt urine samples in a standardized way and to minimize protein loss. Furthermore, the molecular weight cut-off of the ultrafiltration membrane is 3 kDa, leading to removal of low-molecular-weight polypeptides, which are abundant in human urine samples [22,23]. Using the ultrafiltration unit, urine was concentrated about 50-fold. Concentrated protein from single urine sample was separated by one-dimensional sodium dodecyl sulfate (SDS)-polyacrylamide gel electrophoresis (PAGE) and reverse phase high-performance liquid chromatraphy (HPLC). We applied crude concentrates to one-dimensional SDS-PAGE (Figure 2a) and cut the gel into 14 or 10 pieces. Protein mixtures were subjected to in-gel tryptic digestion (in-gel 1 and in-gel 2 subsets). We also applied crude concentrates to a novel macroporous reversed phase column (mRP-C18 high-recovery protein column), but resolution was poor initially (data not shown). We therefore depleted human serum albumin from the urine concentrates using an immuno-affinity column and applied the albumin-depleted protein mixture to the column, resulting in a good resolution with 22 fractions (Figure 2b). Separated proteins were denatured by 2,2,2-trifluoroethanol (TFE) [24,25] or urea and thiourea, and were subsequently digested as described in the Materials and methods section (below; in-solution 1 and in-solution 2 subsets). Concentrated urinary protein from pooled samples was separated by one-dimensional SDS-PAGE, and excised in 10 slices (pool subset). Digests from each set were desalted and concentrated on reversed-phase C_18 _StageTips [26] and analyzed by LC online coupled to electrospray MS.

**Figure 3:**
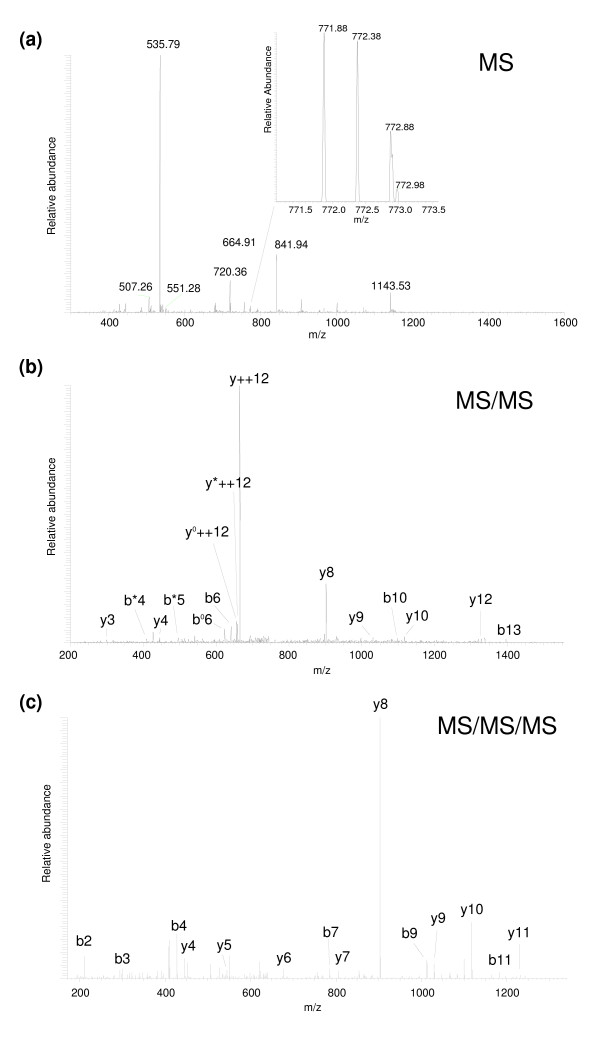
Two consecutive stages of mass spectrometric fragmentation (MS^3^). The precursor of peptide DVPNSQPEMVEAVK **(a**; see insert) was selected for fragmentation from a full scan of mass to charge ratio range. The doubly charged y_12 _fragment ion **(b) **was subsequently fragmented. Characteristic pattern for charged directed fragmentation is observed in MS^3 ^spectra **(c) **and confirms the identification of the above peptide. See Steen and Mann [65] for an introduction to peptide sequencing and confidence of peptide identification. MS, mass spectrometry.

For the single urine sample sets, LC gradients lasted for either 100 or 140 min. The mass spectrometer (LTQ-FTICR) was programmed to perform survey scans of the whole peptide mass range, select the three most abundant peptide signals, and perform SIM scans for high mass accuracy measurements in the FTICR. Simultaneously with the SIM scans, the linear ion trap fragmented the peptide, obtained an MS/MS spectrum, and further isolated and fragmented the most abundant peak in the MS/MS mass spectrum to yield the MS^3 ^spectrum. Figure 3a shows a spectrum of eluting urine peptides. A selected peptide was measured in SIM mode (Figure 3a) and fragmented (MS^2^; Figure 3b). The most intense fragment in the MS/MS spectrum was selected for the second round of fragmentation (Figure 3c). As can be seen in the figure, high mass accuracy, low background level, and additional peptide sequence information obtained from MS^3 ^spectra yielded high-confidence peptide identification. Peak list files obtained from fractions in each subset were merged and the peptide sequences were identified from their tandem mass spectra using a probability based search engine, namely Mascot [27]. Database searches were performed on 15,919, 16,238, 16,312 and 12,180 MS/MS spectra from in-gel 1, in-gel 2, in-solution 1 and in-solution 2, respectively (Table 1). Identified MS^3 ^spectra were automatically scored with in-house developed open source software, MSQUANT [15,28]. As described in Materials and methods (below), proteins were identified using criteria corresponding to a level of false positives of *P *= 0.0005 when at least two peptides were identified, and of *P *= 0.001 when one peptide was identified. We also manually checked MS^2 ^and MS^3 ^spectra for all proteins identified by a single peptide.

To test experimentally the false-positive rate in our dataset, we performed a decoy database search [29]. In this approach peptides are matched against the database containing forward-oriented normal sequences and the same sequences with their amino acid sequences reversed. When requiring the stringent criteria mentioned above, we found no false-positive protein hits. We therefore conclude that our search criteria exclude essentially all false positives.

Using the criteria established here, our analysis of four datasets, two sets employing in-gel digestion and another two sets employing in-solution digestion, resulted in the identification of 8041 unique peptides. In total, 1281 proteins were identified after the removal of contaminants (keratins, trypsin, and endoproteinase Lys-C) and redundant proteins.

For the pooled urine sample, 10 slices from a one-dimensional SDS gel separation were analyzed three times per slice using the LTQ-Orbitrap. A 140 min LC gradient was employed for each analysis. The mass spectrometer was operated in the data-dependent mode. Survey full scan MS spectra (from *m/z *300 to 1600) were acquired in the orbitrap and the most intense ions (up to five, depending on signal intensity) were sequentially isolated and fragmented in the linear ion trap (MS/MS). Peak list files obtained from 10 fractions were processed separately and the peptide sequences were identified as described above. Proteins were identified with criteria corresponding to a level of false positives of *P *= 0.0025 or 1 in 400, which is lower than the total number of proteins in each slice. In this way, independent analysis of the 10 slices allowed us to employ a lower threshold without false-positive identifications, as judged by the decoy database. Altogether, we identified 1055 proteins from 10 slices for the pooled urine sample (Table 2).

**Figure 4:**
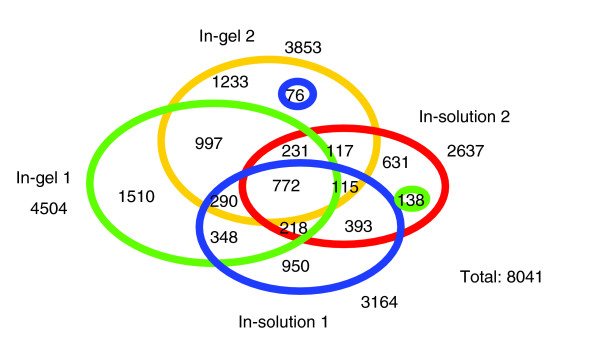
Diagram of peptides found in multiple datasets. All overlaps of peptides are shown (two way, three way, and four way) for all four input datasets: in-gel 1 (green), in-gel 2 (yellow), in-solution 1 (blue), and in-solution 2 (red). Numbers represent the number of shared peptides in the respective overlapping areas.

Of the 8041 peptides identified from urine sample of the single person, 772 (9.6%) were found in all four datasets, 856 (10.6%) were found in three of the four datasets, 2089 (26.0%) were found in two of the four datasets, and the remaining 4324 (53.8%) were found in only one of the four input datasets (Figure 4). Overlaps between in-gel datasets and in-solution datasets were deeper than those between in-gel datasets and an in-solution datasets. Hydrophobicity value of identified peptides in each subset was calculated using the Kyte and Doolittle model [30]. Comparing in-gel specific with in-solution specific peptides, the hydrophobicity values were -0.24 versus -0.54, with an overall hydrophobicity of -0.33 in all datasets. The difference between in-gel and in-solution datasets was not significant but shows the tendency for peptides identified only in in-gel datasets to be more hydrophobic than those identified only in in-solution datasets.

**Figure 5:**
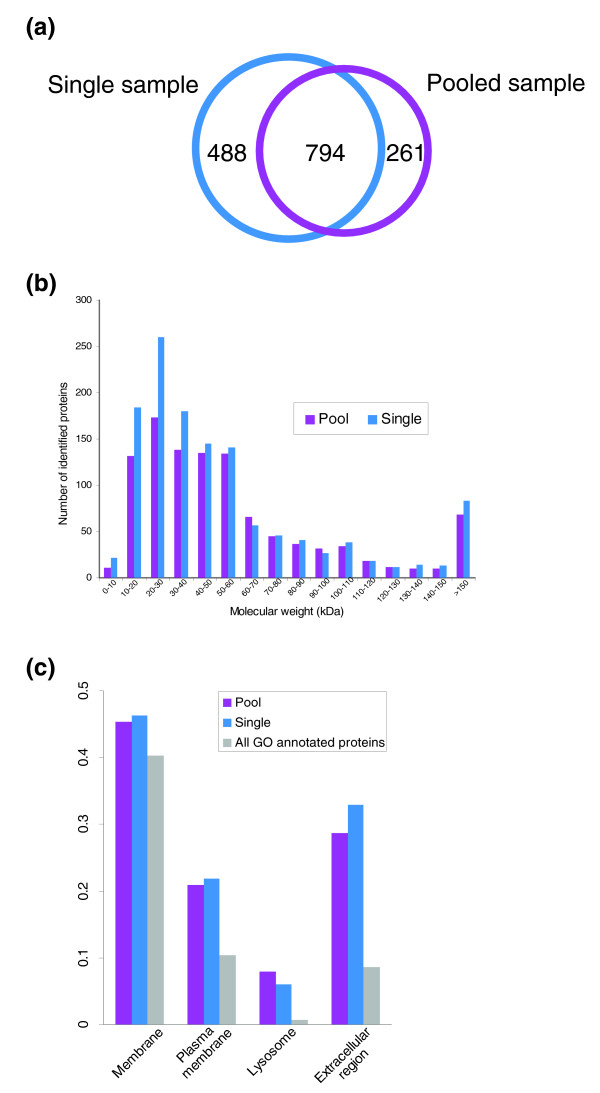
Comparison of identified proteins in urine of a single person and pooled urine from nine persons. **(a) **Overlapping proteins, **(b) **molecular weight distribution, and **(c) **cellular localization were compared. The ratio of membrane, plasma membrane, lysosome, and extracellular region proteins in each dataset were calculated using BiNGO, as described in the Materials and Methods section. GO, Gene Ontology.

As described above the urinary proteome of a single person was investigated in great depth and with different methods. Because the urinary proteome is variable, even from the same individual at different time points, we wished to determine whether the individual urinary proteome was typical. Thus, we compared the overall features of the urinary proteins between single and pooled specimens. As shown in Figure 5, there was deep overlap between the two samples, and the bulk properties in terms of molecular weight and predicted cellular localization were also very similar.

### Characterization of the urinary proteome via Gene Ontology annotation

**Figure 6:**
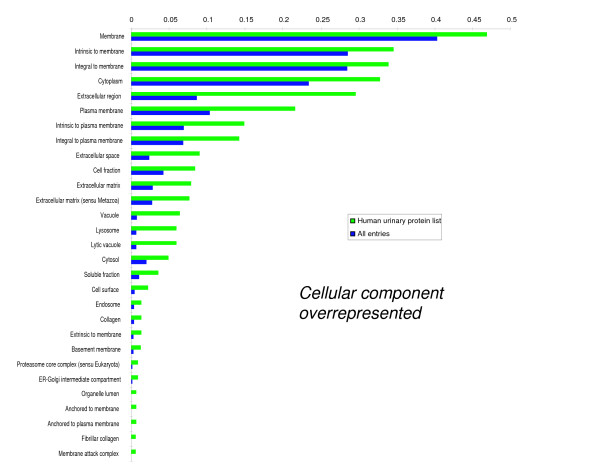
Significantly over-represented GO cellular component terms for the set of identified urinary proteins. The set of identified urinary proteins was compared with the entire list of IPI entries (IPI_Human, version 3.13, 57050 protein sequences), and significantly over-represented and underrepresented GO terms (*P *< 0.001) are shown. The ratio shown is the number of urinary and entire IPI proteins annotated to each GO term divided by the number of urinary and entire IPI proteins linked to at least one annotation term within the indicated GO cellular component, molecular function, and biological process categories. GO, Gene Ontology; IPI, International Protein Index.

**Figure 7:**
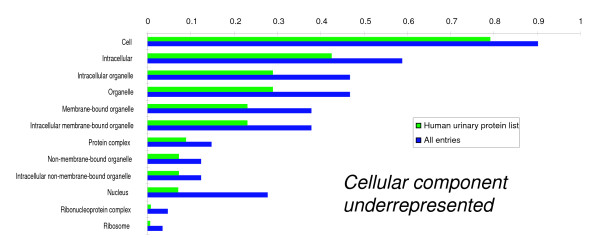
Significantly under-represented GO cellular component, molecular function and biological process terms for the set of identified urinary proteins. Each term was selected as described in the legend to Figure 6. GO, Gene Ontology.

The identified proteins were functionally categorized based on universal Gene Ontology (GO) annotation terms [31] using the Biological Networks Gene Ontology (BiNGO) program package [32,33]. In total, 1041, 1191, and 1118 proteins were linked to at least one annotation term within the GO cellular component, molecular function, and biological process categories, respectively. In total, 214 and 67 terms exhibited significance (*P *< 0.001) as overrepresented and underrepresented terms compared with the entire list of International Protein Index (IPI) entries (IPI_Human, versions 3.13, 57050 protein sequences). As shown in Figures 6 and 7, in the cellular component category, GO terms related to extracellular proteins such as extracellular region (308 proteins found), extracellular space (94), and extracellular matrix (82) were overrepresented, as was expected. In the sample preparation step, we removed cells and debris from the urine by centrifugation, and so GO terms related to intracellular proteins including cell (824), intracellular (442), intracellular organelle (302), nucleus (74), and ribosome (7) were underrepresented. However, unexpectedly, GO terms related to plasma membrane proteins (225) and lysosome proteins (62) were overrepresented. These findings suggest that shed epithelial cells and blood cells are not the main source of the plasma membrane and lysosome proteins identified in our study, but implicate the presence of excretion pathway(s) specific for these proteins.

**Figure 8:**
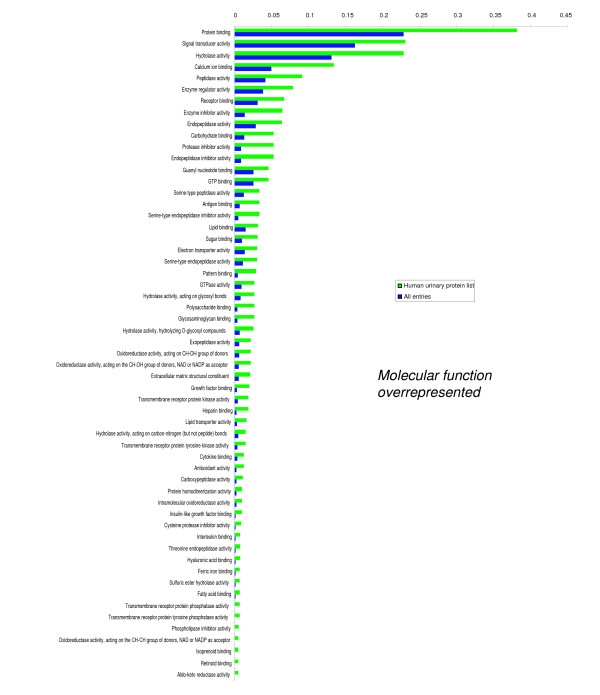
Significantly over-represented GO molecular function terms for the set of identified urinary proteins. Each term was selected as described in the legend for Figure 6. GO, Gene Ontology.

In the molecular function category, 57 GO terms were enriched (Figure 8). Those terms are categorized to four groups: signal transducer, peptidase, enzyme inhibitor, and others. Signal transducer activity (275 proteins found) was unexpected because it was not enriched in an analysis of investigations into a related body fluid, the plasma proteome [34]. Receptor binding (80) is the major subcategory. In particular, growth factor binding (24), including 11 insulin-like growth factor binding proteins, three latent transforming growth factor binding proteins, and five interleukin receptors, was overrepresented. Furthermore, transmembrane receptor protein kinase activity (22) and transmembrane receptor protein tyrosine phosphatase activity (18) were also overrepresented. GTP binding (55) and guanyl nucleotide binding (55) were also enriched terms and shared the same set of proteins, including Ras, Rab, Rho, Arf, and Ras-related proteins.

**Figure 9:**
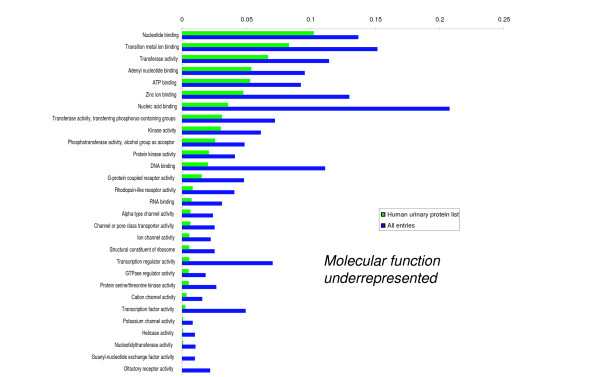
Significantly under-represented GO molecular function terms for the set of identified urinary proteins. Each term was selected as described in the legend of Figure 6. GO, Gene Ontology.

A total of 109 proteins were annotated within the peptidase activity category. Both endopeptidase (76) and exopeptidase (26) activities were overrepresented. We identified 36 serine-type endopeptidases such as kallikreins, thrombins, transmembrane proteases, and nine proteasome subunits. Peptidase inhibitors are necessary to regulate these enzymes, and consequently endopeptidase inhibitor activity (63) was enriched with high significance (*P *< 4.73 × 10^-29^). Of these, 40 proteins belong to the term of serine endopeptidase inhibitor activity. Serine protease inhibitors are important in controlling enzyme activity of activated coagulation factors in the blood. The urinary trypsin inhibitor bikunin (AMBP protein) is among the serine protease inhibitors and is an important anti-inflammatory substance in urine [35]. Extracellular matrix-related terms such as sugar binding, polysaccharide binding, glycosaminoglycan binding, and heparin binding were also overrepresented. In contrast, 29 terms were underrepresented (Figure 9). Most of these were related to intracellular function. DNA binding (24 proteins found) was underrepresented in the urinary proteome; curiously, it was found to be overrepresented in the plasma proteome [34].

**Figure 10:**
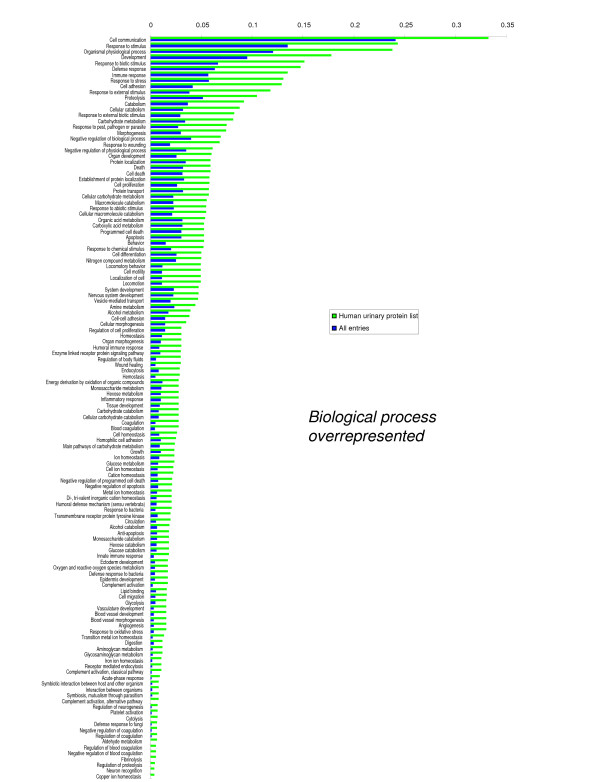
Significantly over-represented GO biological process terms for the set of identified urinary proteins. Each term was selected as described in the legend of Figure 6. GO, Gene Ontology.

**Figure 11:**
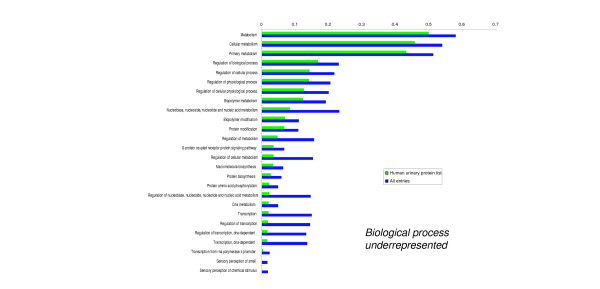
Significantly under-represented GO biological process terms for the set of identified urinary proteins. Each term was selected as described in the legend of Figure 6. GO, Gene Ontology.

Overrepresented and underrepresented GO terms in the biological process category are shown in Figure 10 and 11, respectively. 128 GO terms were enriched and 15 of them were related to immune response (Figure 10). It is reasonable that urine contains many immune response proteins such as chemokines, adhesion molecules, and proinflammatory cytokines because many proteins involved in immune response are known to be present in blood, and the urinary tract is under the same constant threat of infection with intestinal microbiota [36,37]. Enrichment of cell adhesion was the most statistically significant finding (*P *< 4.60 × 10^-32^) in this category. A total of 144 proteins were found in this term and 43 of these proteins belong to cell-cell adhesion, such as cadherins and intracellular adhesion molecules.

## Discussion

### Characteristics of the urinary proteome

**Figure 12:**
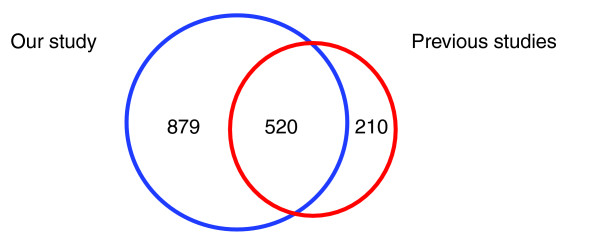
Comparison between proteins identified in the present study and five recently published proteomic datasets.

We identified 1543 proteins in urine from ten healthy donors in this study. Figure 12 shows the overlap of urinary proteins identified in the previous five largest studies [9-13] and our study. In order to compare the different protein identifiers, protein IDs in each dataset were converted to gene symbols using ProteinCenter (Proxeon Bioinformatics, Odense, Denmark). The total sum of unique gene products reported previously is 730. Of those, 520 (71.2%) were also found in our dataset, whereas 210 and 879 gene products were found only in the previous reports or in our study, respectively.

Our study achieved a much higher degree of confidence than did most previous investigations while reporting many more proteins; therefore, the overlap with those studies is surprisingly high. In contrast, previously reported plasma proteomes overlapped barely at all [38].

One of the problems in body fluid proteomics is the tremendous variation in individual protein abundance, which can be as high as 10^10 ^or more in serum and plasma. Thus, depletion of abundant proteins is a standard approach to in-depth analysis of the plasma proteome in the Human Proteome Organization's Plasma Proteome project. In the case of urine, we found this problem to be not as severe. For example, we identified both highly abundant proteins such as serum albumin and low abundance proteins such as growth factors. These proteins span at least three orders of magnitude in concentration, ranging from 1.0-3.3 μg/l (insulin-like growth factor II [39] and platelet-derived growth factor [40]) to 2.2-3.3 mg/l (serum albumin [41]) in normal urine. We concentrated urine samples 50 times, so the concentration of serum albumin in the concentrated sample would be 0.11-0.165 g/l, which is more than 200 times lower than the concentration in plasma (usually 35-50 g/l). The apparently more even distribution of proteins in the urinary proteome makes it possible to identify more than 1000 proteins, a majority of them without depletion of abundant proteins (in-gel samples 1 and 2, and pooled sample).

### Origin of proteins in the urine

Our analysis revealed that extracellular proteins, plasma membrane proteins, and lysosomal proteins are enriched in the urine, whereas other intracellular proteins are not enriched. It was expected that urine would contain many extracellular proteins (by definition); however, the presence of plasma membrane proteins and lysosomal proteins were not expected. These results suggest that there are specific transport pathways for plasma membrane proteins and lysosome proteins.

The excretion pathway of renal apical plasma membrane proteins through the process of exosome formation was previously suggested [42] and was recently demonstrated rigorously using electron microscopy [9]. In our data we identified membrane transporters localized in the kidney. These transporters are involved in water (aquaporin [AQP]1, AQP2, and AQP7), drug (multidrug resistance protein 1), sodium, potassium, and chloride transport (solute carrier family 12 members 1, 2, and 3; sodium/potassium-transporting ATPase gamma chain; potassium voltage-gated channel subfamily E member 3; and amiloride-sensitive sodium channel gamma-subunit [also a copper serum amine oxidase]). These proteins, except potassium voltage-gated channel subfamily E member 3 and amiloride-sensitive sodium channel gamma-subunit, were found in the gel bands that correspond to the molecular weight of the intact forms of these proteins; furthermore, peptides localized in both the extracellular and intracellular regions were detected. Thus, our data strongly suggest that plasma membrane proteins were transported to the urine in an intact form. Furthermore, we identified three aquaporins, namely AQP1, AQP2 and AQP7, which are all aquaporins known to localize to the apical plasma membrane in the kidney, whereas we did not identify any aquaporins that are known to be expressed on the basolateral plasma membrane [43,44]. This finding further supports the notion that the excretion pathway of apical plasma membrane proteins through the process of exosome formation is the dominant pathway and that whole cell shedding plays a minor role. This latter point is also supported statistically by our finding that GO terms related to intracellular 'household' functions are significantly underrepresented in urine. Direct proteomic comparisons of apical and basolateral proteomes would be interesting in this regard [45].

It has been shown that lysosomes can undergo exocytosis [46,47]. This process plays a physiological role in repair of wounds of the plasma membrane and was recently confirmed to occur in mouse primary kidney cells [48]. In this process, stored material in lysosomes was released to the medium (extracellular space), whereas lysosomal membrane protein (LAMP)-1 was shown to be redistributed to the plasma membrane [48]. We identified not only lysosomal enzymes but also lysosomal membrane proteins such as LAMP-1, LAMP-2 and LAMP-3, and lysosomal acid phosphatase. The excretion pathway of these membrane proteins cannot be explained by this lysosomal exocytosis model, but there is a possibility that redistributed lysosomal membrane proteins were excreted through the process of exosome formation.

### Urine as diagnostic material

Urine is clearly a suitable material for the diagnosis of diseases that are related to the kidney and urologic tract. Urine proteome analysis for disease biomarker identification has already been applied to prostate cancer [49], renal cell carcinoma [11,50], bladder cancer [51,52], urothelial carcinoma [53], renal Fanconi syndrome [19], transitional cell carcinoma [54], type 1 diabetes [55], and acute rejection of renal allograft [56,57]. Several biomarker candidates for these diseases have been reported. However, most studies employ two-dimensional gel electrophoresis, and so the identified proteins were limited to soluble and abundant protein classes. In the future it will be necessary to characterize the variation in normal protein concentration levels because the urinary proteome is thought to be variable even from one individual at different time points. If high throughput and quantitative mass spectrometric techniques (for review see [58]) are combined with the methods we employed in the present study, then the rich catalog of urinary proteins now accessible should result in ample opportunity to discover disease biomarkers. In order to facilitate this process, we have made the urinary proteome data accessible at the Max-Planck Unified Proteome database (MAPU) [59].

## Conclusion

Confidence and comprehensiveness are conflicting factors, but employing strategies that achieve very high mass accuracy and two stages of mass spectrometric fragmentation allowed us to establish a high-confidence set of human urinary proteins consisting of 1543 proteins. Our analysis provides the largest and most certain set of proteins present in human urine proteomes and provides a useful reference for comparing datasets obtained using different methodologies. Furthermore, comprehensive GO analysis revealed surprising insights into the physiology of this body fluid, most notably the presence of many membrane proteins. If a quantitative aspect is added [58], then urinary proteomics could contribute to the diagnosis and classification of disease in the future.

## Materials and methods

### Human urine protein concentrates

A single urine sample was obtained from a healthy male individual. A pooled urine sample was collected from nine healthy volunteers who underwent a medical check-up by the doctor of our institute. Personal information on these individuals is given in Additional file 3.

Immediately after urine collection, one protease inhibitor cocktail tablet (Complete™; Roche Diagnostics, Mannheim, Germany) was added per 50 ml urine to avoid proteolysis in the sample, and 5 ml of each sample was pooled together (pooled sample). We also collected a first morning urine sample from a healthy male individual in 100 ml volumes (single sample). These samples were stored on ice prior to centrifugation at 2000 × *g *for 10 min at 4°C. The removal of cells was confirmed by microscopic examination (Additional data file 4). The supernatant was transferred to Centriprep YM-3 membrane concentrators (Millipore, Billerica, MA, USA) and spun at 3000 × *g *to reduce the volumes to about 1 ml for pooled sample and 2 ml for single sample. The protein amounts in urine concentrates were measured using the Coomassie Protein Assay Kit (Pierce, Rockford, IL, USA) and concentrates were frozen at -80°C.

### One-dimensional SDS-PAGE and in-gel digest of human urinary proteins

Protein (150 μg) was applied on a 4-12% Bis-Tris gel (Novex; Invitrogen, Carlsbad, CA, USA) using 2-(N-morpholino)-ethanesulfonic acid or 3-(N-morpholino)propanesulphonic acid SDS running buffer (Invitrogen), in accordance with the manufacturer's instructions. After staining by colloidal Coomassie (Invitrogen), the gel lane was cut into 10 or 14 pieces and subjected to in-gel tryptic digestion, essentially as described by Wilm and coworkers [60]. Briefly, the gel pieces were de-stained and washed, and, after dithiothreitol reduction and iodoacetamide alkylation, the proteins were digested with porcine trypsin (modified sequencing grade; Promega, Madison, WI, USA) overnight at 37°C. The resulting tryptic peptides were extracted from the gel pieces with 30% acetonitrile, 0.3% trifluoroacetic acid (TFA), and 100% acetonitrile. The extracts was evaporated in a vacuum centrifuge to remove organic solvent, and then de-salted and concentrated on self-made reverse phase C18 StageTips, as described previously [26].

### Reverse phase HPLC and in-solution digest of human urinary proteins

Protein (250 μg) was applied to Vivapure Anti-HSA Kit (Vivascience, Hanover, Germany) to deplete serum albumin. Urea and acetic acid were added to the albumin-depleted protein mixture and the final concentrations were adjusted to 6 mol/l and 1.0%, respectively. The albumin-depleted protein mixture was separated on a reverse phase HPLC column (4.6 mm internal diameter × 50 mm long column; mRP-C18 High-Recovery protein column, Agilent Technologies, Palo Alto, CA, USA) at 80°C using linear multi-segment gradient. Following a 10 min wash with 97% solvent A (water in 0.1% TFA) and 3% solvent B (acetonitrile in 0.08% TFA), a linear gradient to 15% solvent B at 12 min, to 35% at 40 min, to 100% at 46 min, to 100% at 51 min, and to 3% at 55 min was achieved using a flow rate of 750 μl/min. Fraction collection was performed by time, collecting 2 min time slices starting at 10 min and continuing to 54 min (total 22 fractions). Each fraction was divided into halves and dried using a vacuum centrifuge and subjected to in-solution tryptic digestion using urea and 2,2,2-trifluoroethanol (TFE; Sigma-Aldrich, St Louis, MO, USA) as a denaturant, respectively.

In-solution digestion using urea was done essentially as described previously by Foster and coworkers [61]. Briefly, fractionated proteins were resolved in a buffer containing 6 mol/l urea and 2 mol/l thiourea, and reduced, alkylated, and digested. To reduce disulfide bonds, 0.5 μg of DTT was added in the protein solutions and incubated for 0.5 hours at room temperature. The free thiol (-SH) groups were subsequently alkylated with 2.5 μg iodoacetamide for 30 min at room temperature in the dark. The reduced and alkylated protein mixtures were digested with 0.5 μg endoproteinase Lys-C (Wako Biochemicals, Osaka, Japan) for 3 hours and with 0.5 μg sequence grade-modified trypsin for overnight at 37°C after dilution to 1.5 mol/l urea with 50 mmol/l NH_4_HCO_3 _(pH 8.0). Proteolysis was quenched by acidification of the reaction mixtures with TFA.

In-solution digestion using TFE was done essentially as described previously by Meza and coworkers [24,25]. Briefly, fractionated proteins were resolved in a buffer containing 50% TFE and reduced, alkylated, and digested. DTT was added to a final concentration of 10 mmol/l in the protein solutions and incubated for 20 min at 90°C. Then, iodoacetamide (50 mmol/l final concentration) was added for alkylation and the solution was incubated for 60 min at room temperature in the dark. Excess iodoacetamide was quenched by DTT (10 mmol/l final concentration) for 60 min at room temperature in the dark. The protein mixtures were diluted to 5% TFE with 20 mmol/l NH_4_HCO_3 _(pH 8.0) and digested with 1.0 μg of sequence grade-modified trypsin for overnight at 37°C. Proteolysis was stopped by acidification with TFA.

Finally, the resulting peptide mixtures were desalted on reverse phase C18 StageTips and diluted in 0.1% TFA for nano-HPLC-MS analysis.

### Nanoflow LC-MS^2 ^or MS^3^

All nanoflow LC-MS/MS and MS^3 ^experiments were performed on a 7-Tesla Finnigan LTQ-FT mass spectrometer and a LTQ-Orbitrap (Thermo Electron, Bremen, Germany) equipped with a nanoelectrospray ion source (Proxeon Biosystems, Odense, Denmark), basically as described previously [15,16,62]. Data were acquired in data-dependent mode using Xcalibur software. In the case of LTQ-FTICR, the precursor ion scan MS spectra (*m/z *300-1575) were acquired in the FTICR with resolution R = 25,000 at *m/z *400 (number of accumulated ions: 5 × 10^6^). The three most intensive ions were isolated and fragmented in the linear ion trap by collisionally induced dissociation using 3 × 10^4 ^accumulated ions. They were simultaneously scanned by FTICR-selected ion monitoring with 10 Da mass range, R = 50000, and 5 × 10^4 ^accumulated ions for even more accurate molecular mass measurements. For MS^3^, the most intense ion with *m/z *above 300 in each MS/MS spectra were further isolated and fragmented. In data-dependent LC-MS/MS experiments, dynamic exclusion was used with 30 s exclusion duration. In the case of the LTQ-Orbitrap, the precursor ion scan MS spectra (*m/z *300-1600) were acquired in the orbitrap with resolution R = 60000 at *m/z *400 with the number of accumulated ions being 1 × 10^6^. The five most intense ions were isolated and fragmented in linear ion trap (number of accumulated ions: 3 × 10^4^). The resulting fragment ions were recorded in the orbitrap with resolution *R *= 15,000 at *m/z *400. The lock mass option enabled accurate mass measurements in both MS and MS/MS mode. The polydimethylcyclosiloxane ions generated in the electrospray process from ambient air (protonated (Si(CH_3_)_2_O)_6_; *m/z *445.120025) were used for internal recalibration in real time. In data-dependent LC-MS/MS experiments dynamic exclusion was used with 30 s exclusion duration.

### Data analysis

Proteins were identified via automated database searching (Mascot; Matrix Science, London, UK) of all tandem mass spectra against an in-house curated version of the Human IPI protein sequence database (IPI version 3.13; 57050 protein sequences [63]) containing all human protein entries from Swiss-Prot, TrEMBL, RefSeq, Ensembl and H-Inv, as well as frequently observed contaminants (porcine trypsin, endoproteinase Lys-C and human keratins). Carbamidomethyl cysteine was set as fixed modification, and oxidized methionine and protein N-acetylation and deamidation of asparagine and glutamine were searched as variable modifications. Initial mass tolerances for protein identification on MS peaks were 3 ppm (LTQ-FT data) and 5 ppm (LTQ-Orbitrap data), and on MS/MS peaks they were 0.5 Da. Two 'missed cleavages' were allowed. The instrument setting for the Mascot search was specified as 'ESI-Trap'. Identified MS^3 ^spectra were automatically scored with MSQUANT (open source software available on the internet [15,28]). Results obtained from Mascot and MSQUANT were imported to our in-house developed peptide-database server, and peptides and proteins were identified using criteria as follows.

For LTQ-FTICR data, only peptides for which the MS^2 ^score was above the 95th percentile of significance (Mascot score > 24) were included. Only fully tryptic peptides with seven amino acids or longer were accepted for identification. Proteins with at least two peptides and a MS^2 ^score of at least 24 (95% significance level) for one of the peptides and at least 31 (99% significance level) for the other were counted as identified protein. For proteins identified by a single peptide, we required the presence of an MS^3 ^spectrum, an MS^2 ^score of at least 34 (99.5% significance level), and a combined score for MS^2 ^and MS^3 ^of above 41 (99.9% significance level) and a peptide delta score (score difference between first and second candidate sequences obtained from a database search) above 5.0. MS^2 ^and MS^3 ^spectra for all proteins identified by a single peptide were manually checked.

For LTQ-Orbitrap data, 10 fractions separated by molecular weight of proteins were analyzed independently. The 95% significance threshold in the database search was a MS^2 ^score of 25 or 26. Proteins were considered positively identified when they were identified with at least two fully tryptic peptides of more than six amino acid length, MS^2 ^score of at least 15 or 16, and a sum of MS^2 ^score of at least 50 or 52 resulting in an expected false-positive rate of 0.25% or 1 in 400.

For counting the number of identified proteins across each experiment, redundant protein identification was removed using Blast search function of ProteinCenter and manual check.

### Enrichment analysis of GO categories

We used BiNGO [32,33] with the Cytoscape plugin to find statistically over- or under-represented GO categories in biologic data as the tool for enrichment analysis of our urinary proteome dataset. For enrichment analysis we needed a test dataset (which is our identified urinary proteome) and a reference set of GO annotation for the complete human proteome. As per instructions on the BiNGO webpage, the custom GO annotation for the reference set (of whole IPI human dataset) was created by extracting the GO annotations available for Human IPI IDs from EBI GOA Human 39.0 release [64]. The GOA Human 39.0 release contains annotations for 28,873 proteins compiled from different sources. The analysis was done using 'hyper geometric test', and all GO terms that were significant with *P *< 0.001 (after correcting for multiple term testing by Benjamini and Hochberg false discovery rate corrections) were selected as over-represented and under-represented.

## Additional data files

The following additional data are included with the online version of this article: An Excel file containing a list of identified proteins in each experiment (Additional data file 1); an Excel file containing a list of the identified peptides in each experiment (Additional data file 2); an Excel file containing personal information on the individuals who provided urine (Additional data file 3); and a pdf file summarizing the results of the microscopic examination to confirm cell removal from urine (Additional data file 4).

## Supplementary Material

Additional data file 1 An Excel file containing a list of identified proteins in each experiment. The spreadsheet consists of 15 worksheets containing respective proteins. Click here for file

Additional data file 2 An Excel file containing a list of the identified peptides in each experiment. The spreadsheet consists of 14 worksheets containing respective peptides. Click here for file

Additional data file 3 An Excel file containing personal information on the individuals who provided urine, showing sample number, age and gender. Click here for file

Additional data file 4 A pdf file summarizing the results of the microscopic examination to confirm cell removal from urine. Click here for file

## Figures and Tables

**Table 1:** Experimental conditions and statistics on database searches of four individual experiments using a single urine sample

	In-gel 1	In-gel 2	In-solution 1	In-solution 2
Urinary protein	150 μg	150 μg	125 μg	125 μg
Albumin removal	-	-	+	+
Protein separation	Invitrogen NuPAGE 4-12% Bis-Tris 1D gel		Agilent mRP-C18 column	
Number of fraction	14	10	22	22
Digestion	In-gel	In-gel	In-solution	In-solution
Denaturant			50% Trifluoroethanol	6 mol/l Urea + 2 mol/l thiourea
LC gradient time	100 min	140 min	100 min	100 min
Identified IT-MS^2 ^spectra by Mascot^a^	16,219	10,535	13,367	10,175
Number of unique peptides^a^	4504	3853	3164	2637
Number of identified proteins^a^	759	815	656	580
Total number of unique peptides^a^	8041			
Total number of identified proteins^a^	1281			

^a^Applied criteria are described in the Materials and methods section. 1D, one-dimensional; LC, liquid chromatography; MS, mass spectrometry.

**Table 2:** Experimental conditions and statistics on database searches of 10 slices of pooled urine sample

	Pooled 1	Pooled 2	Pooled 3	Pooled 4	Pooled 5	Pooled 6	Pooled 7	Pooled 8	Pooled 9	Pooled 10
Protein separation	Invitrogen NuPAGE 4-12% Bis-Tris 1D Gel									
Digestion	In-gel									
LC gradient time	140 min									
Identified IT-MS^2 ^spectra by Mascot	42,578	36,288	46,328	42,664	48,938	46,529	48,101	50,654	26,607	26,817
Number of unique peptides^a^	777	1133	1841	1114	1591	2493	2179	878	1671	2006
Number of identified proteins^a^	125	186	290	186	229	302	239	96	206	153
Total number of unique peptides^a^	9737									
Total number of identified proteins^a^	1055									

^a^Applied criteria are described in the Materials and methods section.
