# Supplementary material for: The human urinary proteome contains more than 1500 proteins, including a large proportion of membrane proteins
Source: Genome Biol. 2006 Sep 1;7(9):R80. doi: 10.1186/gb-2006-7-9-r80 (PMC1794545; doi:10.1186/gb-2006-7-9-r80)
Supplement: Additional data file 4 — A pdf file summarizing the results of the microscopic examination to confirm cell removal from urine. [file gb-2006-7-9-r80-S4.pdf]

Removal of cells in the urine by centrifugation

(a)

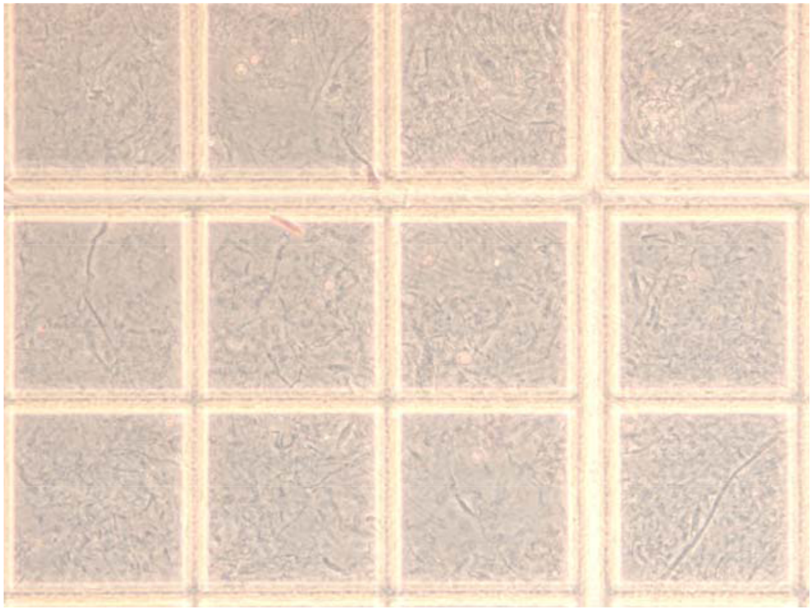

(b)

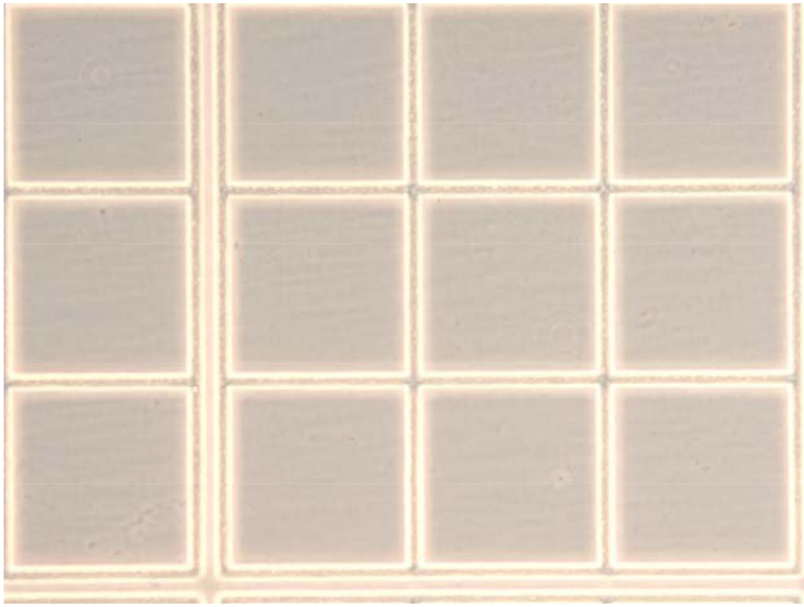

(c)

	crude (cell number / µL)	supernatant (cell number / µL)
sample 1	2.1±0.7	0.0±0.0
sample 2	8.4±0.9	0.0±0.0
sample 3	1.1±0.2	0.1±0.1

Confirmation of cell removal by microscopic examination. (a) 10 mL of the urine was centrifuged at 5000 g for 30 min. Following centrifugation, supernatant was carefully removed and final urine volume with sediment was adjusted to 190 µL. Urine sediment was stained by 10 µL of KOVA STAIN (Hycor biomedical, Kassel, Germany) and a number of cells in the sediment was counted using KOVA glasstic slide10 (Hycor) and an inverted microscope (Leica DM IRB & DFC480, Wetzlar, Germany). Images were captured using low-power magnification (Total Magnification: 100 X). **(b)** 12 mL of the urine was centrifuged at 2000 g for 10 min. 10 mL of the supernatant was centrifuged again at 5000 g for 30 min. Following centrifugation, urine sediment was stained and cell number was counted as described above. **(c)** Three urine samples were used to count cells in the urine sediment. Experiments were carried out in triplicate. Data are means±SD of one triplicate experiment.
